# Efficacy of a Saffron-Based (*Crocus sativus* L.) Herbal Supplement on Sleep Disturbances in Pulmonary Sarcoidosis: A Randomized Single-Blind Controlled Trial

**DOI:** 10.5812/ijpr-169041

**Published:** 2026-06-08

**Authors:** Zahra Moghimi Dehkordi, Jamshid Salamzadeh, Maria Tavakoli-Ardakani, Atefeh Abedini, Farzaneh Dastan

**Affiliations:** 1 Department of Clinical Pharmacy, School of Pharmacy, Shahid Beheshti University of Medical Sciences, Tehran, Iran; 2 Chronic Respiratory Diseases Research Center (CRDRC), National Research Institute of Tuberculosis and Lung Diseases (NRITLD), Shahid Beheshti University of Medical Sciences, Tehran, Iran; 3 Department of Pharmacy, Monash Health, Melbourne, Australia

**Keywords:** Crocus Sativus, Saffron, Insomnia, Quality Of Life, Sarcoidosis, Pulmonary, Phytotherapy

## Abstract

**Background:**

Sleep disturbances in pulmonary sarcoidosis increase fatigue and reduce quality of life; however, standard anti-inflammatory treatments provide limited benefit. Saffron (*Crocus sativus* L., family Iridaceae), which has anti-inflammatory and neuromodulatory properties, may improve sleep, although its effects in this population have not been studied.

**Objectives:**

This study evaluated whether 60 days of saffron supplementation (30 mg twice daily) improves sleep quality, reduces daytime sleepiness and fatigue, and enhances quality of life in patients with pulmonary sarcoidosis.

**Methods:**

In this single-blind randomized controlled trial, 76 adults with pulmonary sarcoidosis, confirmed according to the American Thoracic Society/European Respiratory Society/World Association of Sarcoidosis and Other Granulomatous Disorders criteria, and Pittsburgh Sleep Quality Index (PSQI) scores ≥ 5 were assigned to groups using permuted block randomization (block size, 4). Allocation concealment was ensured using opaque, sealed, sequentially numbered envelopes. Participants were randomly allocated to receive either saffron (30 mg twice daily) plus sleep-hygiene counseling or counseling alone for 60 days. Sleep quality, daytime sleepiness, fatigue, and quality of life were assessed at baseline and day 60 using the PSQI, General Sleep Disturbance Scale (GSDS), Epworth Sleepiness Scale (ESS), Fatigue Assessment Scale (FAS), Patient-Reported Outcomes Measurement Information System (PROMIS)-fatigue, and 12-item Short Form Health Survey (SF-12).

**Results:**

Of the 76 participants enrolled, 72 completed the study (36 per group). Compared with the control group, saffron supplementation significantly improved PSQI (adjusted mean difference [MD], -2.21), GSDS (adjusted MD, -14.60), ESS (adjusted MD, -2.46), FAS (adjusted MD, -4.58), and the PROMIS global physical health score (adjusted MD, 1.65) (all P < 0.001). Significant improvements were also observed in the SF-12 physical (P = 0.03) and mental (P = 0.003) component scores. No serious adverse events occurred.

**Conclusions:**

Adjunctive saffron supplementation significantly improved sleep quality, reduced daytime sleepiness and fatigue, and enhanced quality of life in patients with pulmonary sarcoidosis. Larger, placebo-controlled studies are warranted to confirm these findings.

## 1. Background

Sarcoidosis is a systemic inflammatory disorder characterized by granuloma formation in multiple organs, with pulmonary involvement being the most common manifestation (1, 2). Pulmonary sarcoidosis often presents with symptoms such as cough, dyspnea, and fatigue. Sleep disturbances, including excessive daytime sleepiness, poor sleep quality, and altered sleep architecture, are common in patients with pulmonary sarcoidosis. These disturbances exacerbate fatigue, impair physical function, and reduce overall quality of life (3-5). Several factors contribute to sleep disorders in this population, including respiratory symptoms associated with pulmonary sarcoidosis (3), granulomatous inflammation of the upper airway (6), weight gain associated with corticosteroid use (7), and possible hypothalamic involvement in neurosarcoidosis, which disrupts sleep and circadian rhythm regulation (8).

**Figure 1.**
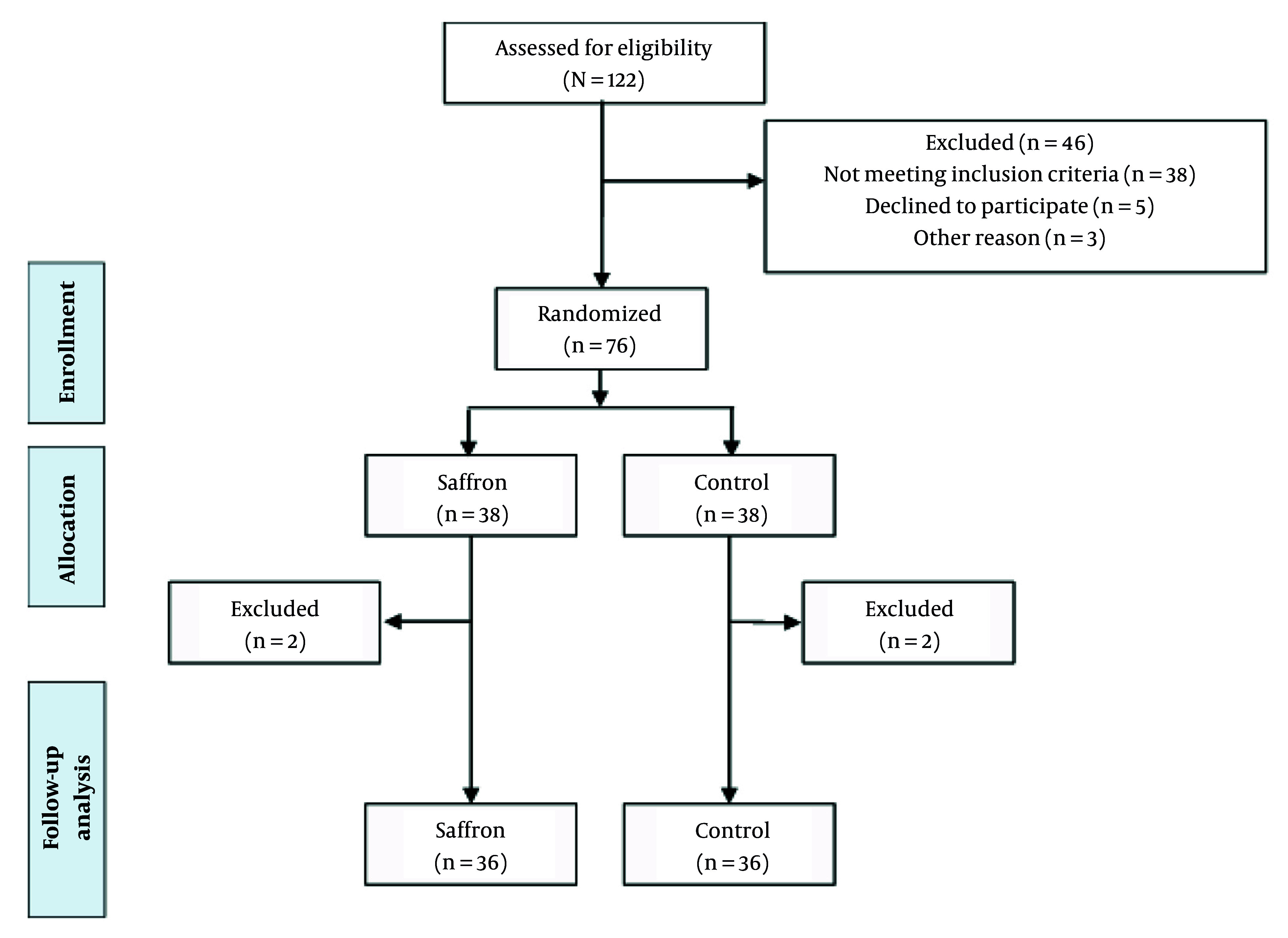
*Crocus sativus* L. (saffron) flower

Standard treatments, such as corticosteroids and immunosuppressive therapies, reduce inflammation but may cause adverse effects, including fatigue, mood changes, and sleep disturbances (9). Consequently, interest is increasing in alternative therapies that may relieve symptoms without these drawbacks. Saffron, the dried stigmata of *Crocus sativus* L. (Iridaceae), is a well-known medicinal plant (Figure 1). Its pharmacological profile is mainly attributed to key bioactive phytochemicals, including crocin, crocetin, picrocrocin, and safranal. In addition to its anti-inflammatory, antioxidant, and mood-regulating properties (10-12), saffron may improve sleep by modulating γ-aminobutyric acid-mediated and serotonergic systems.

Saffron’s potent anti-inflammatory and antioxidant effects make it a particularly relevant intervention for sarcoidosis. Sarcoidosis is driven by systemic inflammation and elevated pro-inflammatory cytokines such as tumor necrosis factor α and interleukin 6, which also contribute to sleep dysfunction. Saffron’s ability to downregulate these inflammatory pathways (13) suggests targeted therapeutic potential for this patient group beyond general sedation. Studies have shown that saffron can improve insomnia and sleep quality in healthy individuals and in those with depression or anxiety (14, 15). However, its effects on sleep disturbances in patients with pulmonary sarcoidosis remain largely unstudied, leaving an important gap in the literature.

## 2. Objectives

This study evaluated the effects of a saffron-based supplement on sleep quality, daytime sleepiness, and fatigue in patients with pulmonary sarcoidosis. Using validated tools, including the PSQI, GSDS, ESS, FAS, PROMIS, and SF-12, it assessed saffron’s therapeutic potential over a 60-day intervention period and aimed to provide evidence supporting a novel complementary treatment to improve quality of life in patients with pulmonary sarcoidosis.

## 3. Methods

### 3.1. Study Design

This randomized controlled trial used a single-blind design and was conducted at Masih Daneshvari Hospital, a university-affiliated, high-volume teaching hospital in Tehran, Iran. Over a one-year period, adult patients with clinically and radiologically confirmed pulmonary sarcoidosis and sleep disturbances were enrolled. After eligibility screening and a detailed explanation of the study protocol, participants were randomized to the saffron or control group and followed for a 60-day treatment period. Participants maintained stable baseline pharmacological treatments for pulmonary sarcoidosis, and no new medications for sleep management were permitted during the trial, ensuring a clear evaluation of the effects of the saffron supplement.

### 3.2. Ethical Considerations

The study protocol was approved by the Ethics Committee of Shahid Beheshti University of Medical Sciences (IR.SBMU.PHARMACY.REC.1403.298). The trial was registered in the Iranian Registry of Clinical Trials (IRCT20250224064834N1) and complied with national regulations. The study was conducted in accordance with the CONSORT 2010 guidelines for reporting randomized trials. All procedures adhered to the Declaration of Helsinki, and participants provided written informed consent before any study-related assessments or interventions.

### 3.3. Recruitment

Between late April 2025 and September 2025, consecutive adult outpatients and inpatients at the pulmonary sarcoidosis clinic of Masih Daneshvari Hospital were screened. Investigators reviewed medical records to identify patients aged 18 years or older with pulmonary sarcoidosis confirmed by expert pulmonologists according to the American Thoracic Society/European Respiratory Society/World Association of Sarcoidosis and Other Granulomatous Disorders clinical practice guidelines (16). The diagnosis was established based on a combination of clinical presentation, radiological findings (eg, bilateral hilar lymphadenopathy on chest X-ray or computed tomography), and histological evidence of noncaseating granulomas, while excluding other potential causes of granulomatous disease. All identified patients then completed the PSQI to confirm significant sleep disturbance (PSQI global score ≥ 5). Eligible patients were invited to participate, and those who consented underwent a comprehensive baseline assessment.

### 3.4. Eligibility Criteria

Participants were required to be 18 years or older, have a confirmed diagnosis of pulmonary sarcoidosis based on the aforementioned international criteria, have significant sleep disturbance defined as a PSQI global score ≥ 5 (17), and be able to provide informed consent. Exclusion criteria were pregnancy or lactation; a history of central nervous system disorders, such as stroke, epilepsy, or brain tumors; use of sedative-hypnotic or stimulant medications within two weeks; ongoing anticoagulant therapy; adrenal insufficiency; known allergy to saffron or its formulation; parasomnia or shift-work sleep disorder; fluvoxamine use; diagnosed psychiatric disorders or substance abuse; excessive consumption of alcohol (> 3 standard drinks/day), tea (> 500 mL/day), or energy drinks (> 250 mL/day); and clinically significant tachycardia.

### 3.5. Randomization and Blinding

A block randomization scheme with 19 blocks of 4 patients each ensured balanced 1:1 allocation. Within each block, 2 patients were assigned to the intervention group and 2 to the control group. To maintain blinding, outcome assessors and data analysts were unaware of group assignments until data collection and locking were complete. Participants were aware of their treatment assignment (saffron or counseling alone) because of the nature of the intervention. The allocation sequence was concealed from all personnel involved in recruitment and assessment using sequentially numbered, opaque, sealed envelopes, which were opened only at the time of assignment.

### 3.6. Interventions

The saffron group received a 30-mg capsule of standardized dry extract of *Crocus sativus* L. (DuraLife Saffron, Faranchimi Pharmaceutical Company, Tehran, Iran) twice daily (total dose, 60 mg/day) for 60 days. To ensure consistency, all capsules were obtained from a single production batch. The inactive excipients were microcrystalline cellulose, magnesium stearate, and silicon dioxide. This dosage was selected based on clinical evidence suggesting that 60 mg/day provides enhanced therapeutic effects in chronic inflammatory conditions while maintaining safety (18, 19). In addition, a 60-day (8-week) duration was selected to allow sufficient time for modulation of inflammatory markers and stabilization of sleep-wake patterns, consistent with previous clinical trials in chronic conditions (20, 21). The use of a standardized extract rather than crude powder ensured consistent delivery of bioactive compounds. The extract was standardized according to ISO 3632 criteria and contained a minimum of 2% safranal. A Certificate of Analysis was provided by the manufacturer to confirm the purity and chemical profile of the extract.

Both groups received identical sleep-hygiene counseling based on Centers for Disease Control and Prevention guidelines (22). The control group continued usual care without additional herbal supplements. Participants kept daily sleep-wake diaries, and study staff monitored adherence regularly. No rescue sedative-hypnotic medications were permitted; any participant requiring such intervention for symptom management would have been withdrawn from the study to maintain data integrity. No participant required or reported the use of rescue medication during the trial. Safety monitoring was performed throughout the 60-day period. A blinded investigator conducted weekly telephone follow-ups to monitor treatment adherence and prospectively screen for potential adverse events using a structured checklist. Participants were also instructed to report any unexpected symptoms immediately.

### 3.7. Sample Size

The sample size was determined based on the mean difference and standard deviation of the PSQI reported in the randomized clinical trial by Feizabadi et al. on sleep disorders in patients with pulmonary sarcoidosis (23). Assuming a clinically meaningful between-group difference of 3.0 points and a standard deviation of 4.0 for sleep quality, a total of 28 participants in each group was required to detect a 3-point difference in the PSQI global score with 80% power and a two-sided type I error rate of 0.05. To account for an anticipated dropout rate of approximately 35%, final enrollment was increased to 38 participants per group (total N = 76), providing sufficient statistical power to detect meaningful differences between the saffron and control groups.

### 3.8. Study Outcomes

The primary outcome was the change in sleep quality from baseline to the end of the 60-day intervention, measured by the PSQI global score. Secondary outcomes included changes in daytime sleepiness (ESS), fatigue (FAS), sleep-related symptoms (GSDS), and health-related quality of life, assessed using the PROMIS and SF-12.

### 3.9. Outcome Measurement

Sleep quality was measured using the PSQI and GSDS (17, 24). The PSQI assesses seven sleep dimensions, including latency, subjective sleep quality, efficiency, duration, disturbances, daytime dysfunction, and use of sleep medication, yielding a global score from 0 to 21, with higher scores indicating worse sleep quality (17, 25). The GSDS is a 21-item tool that measures the frequency and severity of sleep disturbances across multiple domains, with total scores indicating disturbance frequency from none to frequent (26, 27).

Daytime sleepiness was evaluated using the ESS. Participants rated their likelihood of dozing in eight daily situations from 0 (never) to 3 (high chance). ESS scores range from 0 to 24, with scores ≥ 11 indicating excessive daytime sleepiness (28).

Fatigue was assessed using the FAS and the PROMIS fatigue module. The FAS includes 10 statements rated on a Likert scale from 1 (never) to 5 (always), with scores ≥ 21 indicating significant fatigue (29). The PROMIS fatigue module measures fatigue severity and impact, with higher scores reflecting a greater fatigue burden.

Health-related quality of life was measured using the SF-12, which produces Physical Component Summary (PCS-12) and Mental Component Summary (MCS-12) scores standardized to a mean of 50 (SD = 10), with higher scores indicating better physical or mental health (30, 31).

All outcomes were assessed at baseline (day 1) and at the end of the 60-day intervention.

### 3.10. Follow-up

Weekly telephone contacts reinforced sleep hygiene, confirmed adherence to the saffron regimen or usual care, and recorded adverse events or concomitant medications. Participants with intolerable side effects or clinical worsening were evaluated by the study physician and could withdraw or receive appropriate treatment. Final in-clinic assessments were performed at the end of the 60-day period, and all outcome measures were re-administered.

### 3.11. Statistical Analysis

Data were analyzed using SPSS software version 26.0 (IBM Corp., Armonk, NY, USA). The Shapiro-Wilk test was used to assess the normality of continuous variables. Normally distributed data were reported as means ± standard deviations (SDs). To account for baseline imbalances and ensure robust comparisons, the primary analysis of between-group differences was performed using analysis of covariance (ANCOVA), with postintervention scores as the dependent variable, study group as the fixed factor, and baseline scores as the covariate. Paired t-tests were used to assess within-group changes. Non-normally distributed variables were summarized as medians with interquartile ranges and analyzed using the Mann-Whitney U test. Categorical data were presented as frequencies and percentages and compared using the chi-square test. A two-tailed P value < 0.05 was considered statistically significant.

Statistical inference was prioritized for the primary outcome (PSQI). To address multiplicity, analyses of secondary outcomes were considered exploratory. Therefore, interpretation focused on adjusted mean differences, effect sizes, and 95% confidence intervals (CIs), rather than relying solely on P values, to minimize the risk of type I error. Data were analyzed using a completer (complete-case) approach, including all participants who completed postintervention assessments. Of the 76 randomized participants (38 per group), 4 (5.3% overall; 2 per group) withdrew before study completion and did not provide final data. Because attrition was low and balanced between groups, the risk of attrition bias was considered minimal, and the final analysis included the 72 participants (36 per group) who completed the study.

## 4. Results

The study initially screened 122 patients with pulmonary sarcoidosis. After 46 patients were excluded because they did not meet the inclusion criteria or declined participation, 76 patients were randomized into 2 groups. During the trial, 4 participants (2 in each group) withdrew. Consequently, 72 patients completed the trial, including 36 in the intervention group and 36 in the control group. Table 1 summarizes the participants’ demographic characteristics. Both groups received standard immunosuppressive treatments, with prednisolone being the most commonly used medication. There were no significant differences in body mass index (BMI), sex, or baseline disease severity between groups (P > 0.05).

**Table 1.** Participant Demographics and Baseline Clinical Characteristics ^a, b^

Characteristics	Saffron (n = 36)	Control (n = 36)	P-Value
**Gender**			0.79
Male	9 (25.0)	10 (27.7)	
Female	27 (75.0)	26 (72.2)	
**Age (y)**	52.64 ± 8.65	54.53 ± 7.73	0.33
**BMI (kg/m^2^)**	24.91 ± 3.00	25.11 ± 1.41	0.72
**Scadding stage**			0.85
0	1 (2.8)	2 (5.6)	
I	20 (55.6)	22 (61.1)	
II	12 (33.3)	10 (27.8)	
III	3 (8.3)	2 (5.6)	
IV	0 (0.0)	0 (0.0)	
**Medications**			
Methotrexate	22 (61.1)	19 (52.8)	0.63
Prednisolone	32 (88.9)	31 (86.1)	1.00
Hydroxychloroquine	13 (36.1)	10 (27.8)	0.61
Mycophenolate mofetil	3 (8.3)	4 (11.1)	1.00
**Past medical history**			
Hypothyroidism	2 (5.6)	1 (2.8)	1.00
Anemia	2 (5.6)	4 (11.1)	0.67
Diabetes	4 (11.1)	2 (5.6)	0.67

^a^ Values are expressed as No. (%) or mean ± SD. Abbreviation: BMI, Body Mass Index.

^b^ Scadding stage 0, no chest abnormality; stage I, hilar lymphadenopathy; stage II, hilar lymphadenopathy with parenchymal abnormality; stage III, parenchymal abnormality without hilar lymphadenopathy; stage IV, fibrosis with volume loss.

As shown in Table 1, there were no significant differences between the saffron and control groups in demographic characteristics, baseline clinical status (including Scadding stage), or background medications (P > 0.05). Prednisolone was the most frequently prescribed medication in both groups (88.9% in the saffron group vs 86.1% in the control group).

To evaluate fatigue, quality of life, and sleep quality, the PROMIS Global Health physical and mental subscales, FAS, SF-12 PCS and MCS, PSQI, ESS, and GSDS were used. Table 2 presents baseline values for the saffron and control groups. At baseline, no significant differences were observed between the groups (P > 0.05), except for the GSDS score (P = 0.04).

**Table 2.** Baseline Scores of the Study Outcomes ^a^

Score	Saffron	Control	Mean Difference (95% CI)	P-Value
**FAS**	33.00 ± 5.17	32.00 ± 4.37	1.00 (-1.25 to 3.25)	0.38
**PROMIS Global Physical Health Raw Score**	12.33 ± 2.38	11.97 ± 3.19	0.36 (-0.96 to 1.68)	0.59
**PROMIS Global Mental Health Raw Score**	11.89 ± 2.49	11.72 ± 3.61	0.17 (-1.30 to 1.63)	0.82
**PCS-12**	35.33 ± 5.75	36.56 ± 9.83	-1.23 (-5.04 to 2.57)	0.52
**MCS-12**	38.35 ± 7.47	40.31 ± 9.93	-1.95 (-6.09 to 2.19)	0.35
**PSQI**	11.56 ± 3.38	10.56 ± 4.59	1.00 (-0.90 to 2.90)	0.30
**ESS**	8.39 ± 4.96	6.97 ± 4.24	1.42 (-0.75 to 3.59)	0.20
**GSDS**	57.33 ± 10.72	50.81 ± 15.57	6.53 (0.23 to 12.83)	0.04 ^b^

^a^ Values are expressed as mean ± SD unless indicated. Abbreviations: CI, confidence interval; ESS, Epworth Sleepiness Scale; FAS, Fatigue Assessment Scale; GSDS, General Sleep Disturbance Scale; MCS-12, mental component summary; PCS-12, physical component summary; PROMIS, patient-reported outcomes measurement information system; PSQI, Pittsburgh Sleep Quality Index.

^b^ Statistically significant at P < 0.05.

The PSQI was used to measure the primary outcome of sleep quality. At baseline, the mean ± SD PSQI scores were 11.56 ± 3.38 in the saffron group and 10.56 ± 4.59 in the control group (P = 0.30; Table 2). All participants (100%) had PSQI scores ≥ 5, indicating poor sleep quality at study entry. After the 60-day intervention, the saffron group had a significantly lower adjusted mean ± SE PSQI score than the control group (8.40 ± 0.31 vs 10.61 ± 0.31; P < 0.001; Table 3). Similarly, daytime sleepiness, as measured by the ESS, was significantly reduced in the saffron group compared with the control group (adjusted mean ± SE, 4.20 ± 0.30 vs 6.66 ± 0.30; P < 0.001; Table 3).

**Table 3.** Between-Group and Within-Group Comparisons of Variations in Study Outcomes ^a^

Score	Saffron	Control	P-value (ANCOVA, Between Groups)	Adjusted Mean Difference (95% CI) ^b^	Effect Size (ηp^2^)
**FAS**			< 0.001 ^c^	-4.58 (-5.86 to -3.29)	0.42
Adjusted	26.04 ± 0.45	30.62 ± 0.45			
P-value (within groups)	< 0.001 ^c^	0.001 ^c^			
**Global Physical Health Raw Score**			< 0.001 ^c^	1.65 (1.08 to 2.22)	0.33
Adjusted	12.55 ± 0.20	10.90 ± 0.20			
P-value (within groups)	0.06	< 0.001 ^c^			
**Global Mental Health Raw Score**			0.09	0.50 (-0.07 to 1.06)	0.04
Adjusted	11.46 ± 0.20	10.96 ± 0.20			
P-value (within groups)	0.08	0.001 ^c^			
**PCS-12**			0.03 ^c^	2.17 (0.21 to 4.14)	0.07
Adjusted	38.95 ± 0.69	36.77 ± 0.69			
P-value (within groups)	< 0.001 ^c^	0.358			
**MCS-12**			0.003 ^c^	3.99 (1.44 to 6.54)	0.12
Adjusted	44.66 ± 0.90	40.67 ± 0.90			
P-value (within groups)	< 0.001 ^c^	0.13			
**PSQI**			< 0.001 ^c^	-2.21 (-3.09 to -1.33)	0.27
Adjusted	8.40 ± 0.31	10.61 ± 0.31			
P-value (within groups)	< 0.001 ^c^	0.30			
**ESS**			< 0.001 ^c^	-2.46 (-3.32 to -1.61)	0.32
Adjusted	4.20 ± 0.30	6.66 ± 0.30			
P-value (within groups)	< 0.001 ^c^	0.001 ^c^			
**GSDS**			< 0.001 ^c^	-14.60 (-17.23 to -11.97)	0.64
Adjusted	36.59 ± 0.92	51.19 ± 0.92			
P-value (within groups)	< 0.001 ^c^	< 0.001 ^c^			

^a^ Values are expressed as mean ± SE unless indicated. Abbreviations: Adj, adjusted; CI, confidence interval; ESS, Epworth Sleepiness Scale; FAS, Fatigue Assessment Scale; GSDS, General Sleep Disturbance Scale; MCS-12, mental component summary; PCS-12, physical component summary; PSQI, Pittsburgh Sleep Quality Index; SE, standard error.

^b^ P values represent between-group comparisons at follow-up using ANCOVA, with baseline scores included as covariates.

^c^ Statistically significant at P < 0.05.

For the secondary outcomes, exploratory analyses showed significant improvements in the saffron group across all measures. Specifically, GSDS scores decreased substantially. At baseline, the saffron group reported greater sleep disturbances than the control group (57.33 ± 10.72 vs 50.81 ± 15.57; P = 0.04; Table 2). After adjustment for baseline differences using ANCOVA, the saffron group demonstrated significantly greater improvement in GSDS scores than the control group (P < 0.001; Table 3). This improvement was particularly evident in items related to difficulty initiating sleep, nighttime awakenings, and early morning wakefulness.

Other secondary outcomes, including daytime sleepiness, fatigue, and quality of life, also showed significant improvements. The adjusted mean ESS score at 60 days was significantly lower in the saffron group than in the control group (4.20 ± 0.30 vs 6.66 ± 0.30; P < 0.001; Table 3). Although both groups showed within-group improvements, the saffron group had a significantly greater reduction in ESS score than the control group (mean difference, -2.46; 95% CI, -3.32 to -1.61).

Fatigue and health-related quality of life also improved. FAS scores were significantly lower in the saffron group at the end of the study than in the control group (adjusted mean, 26.04 ± 0.45 vs 30.62 ± 0.45; P < 0.001). Furthermore, the saffron group showed significantly higher adjusted scores in the SF-12 summary components, including the PCS-12 (38.95 ± 0.69 vs 36.77 ± 0.69; P = 0.03) and MCS-12 (44.66 ± 0.90 vs 40.67 ± 0.90; P = 0.003), compared with the control group. For the PROMIS Global Health scales, the saffron group demonstrated a significant improvement in global physical health (12.55 ± 0.20 vs 10.90 ± 0.20; P < 0.001), whereas a positive but nonsignificant trend was observed for global mental health (11.46 ± 0.20 vs 10.96 ± 0.20; P = 0.09; Table 3).

### 4.1. Adverse Events

Overall, the saffron supplement was well tolerated. Only 1 participant (2.8%) in the saffron group reported a mild, self-resolving headache, and no serious adverse events occurred during the study. There were no study discontinuations due to adverse events in either group (Table 4).

**Table 4.** Adverse Events in the Study Groups ^a^

Adverse Event	Saffron (n = 36)	Control (n = 36)	P Value
**Any adverse event**	1 ^b^ (2.8)	0 (0.0)	0.99
**Serious adverse events**	0 (0.0)	0 (0.0)	-
**Discontinuation due to adverse events**	0 (0.0)	0 (0.0)	-

^a^ Values are expressed as No. (%). Abbreviation: AEs, adverse events.

^b^ One participant reported a mild, self-resolving headache.

## 5. Discussion

Saffron (*Crocus sativus* L.), a natural herbal remedy, has attracted attention for its therapeutic potential in mood disorders and sleep-related problems. Studies suggest that saffron may be a promising alternative to synthetic supplements such as melatonin, with minimal side effects (32). The mechanisms underlying fatigue, poor sleep quality, and excessive daytime sleepiness in patients with sarcoidosis remain unclear. These symptoms are common and substantially impair quality of life, likely due to multiple factors, including respiratory symptoms of pulmonary sarcoidosis, granulomatous inflammation of the upper airway, corticosteroid-related weight gain, and neurosarcoidosis involvement. Saffron’s antioxidant and anti-inflammatory properties have been shown to improve sleep quality and reduce fatigue in various studies, possibly through modulation of the central nervous system and enhancement of serotonergic pathways, similar to the effects of melatonin (6-8, 11, 14, 15, 33). In addition, recent evidence suggests that saffron’s multi-target properties may specifically benefit inflammatory conditions such as pulmonary sarcoidosis.

Sleep disturbances affect a large proportion of patients with sarcoidosis, with 67% reporting poor sleep quality (global PSQI score > 5) (34). These disturbances, independent of disease severity, contribute to increased fatigue, depression, cognitive impairment, and reduced quality of life, as reported by Benn et al. (26). A randomized double-blind trial by Pachikian et al. (35) found that saffron extract (15.5 mg daily for 6 weeks) significantly improved sleep quality in patients with chronic primary insomnia, based on the Leeds Sleep Evaluation Questionnaire and PSQI scores. Similarly, Milajerdi et al. (20) reported that saffron supplementation reduced anxiety, depression, and sleep disturbances in patients with type 2 diabetes. Systematic reviews and meta-analyses (14, 33), further confirm saffron’s effectiveness in improving sleep duration and quality compared with placebo.

In the present study, the saffron group demonstrated a clinically significant within-group improvement in sleep quality, with PSQI scores decreasing by 3.16 points (from 11.56 to 8.40), exceeding the established minimal clinically important difference of 3 points. Furthermore, ANCOVA revealed a significant between-group adjusted mean difference of -2.21 points (95% CI, -3.09 to -1.33; P < 0.001) in favor of the saffron group. These findings indicate that the therapeutic effect of saffron was robust enough to yield a statistically superior and clinically perceptible outcome. In a clinical trial, Akhondzadeh Basti et al. (21) compared saffron capsules (15 mg twice daily) with fluoxetine (10 mg twice daily) in patients with depression and found comparable effects after 8 weeks. In addition, Ashtiani et al. (19) showed that saffron syrup significantly reduced fatigue in patients with multiple sclerosis, supporting its potential for fatigue management.

Limited data are available on the management of fatigue in sarcoidosis, and even effective treatments for active sarcoidosis do not fully relieve the severe fatigue that often accompanies it (36). In this context, the present study provides important insights. The FAS is the only fatigue scale designed specifically for sarcoidosis. Our findings showed a significant between-group difference of -4.58 points (95% CI, -5.86 to -3.29; P < 0.001) in adjusted FAS scores. This demonstrates that the observed reduction in fatigue was statistically significant and clinically perceptible to patients.

The positive impact of saffron on respiratory-related outcomes observed in this study is consistent with findings in other chronic pulmonary conditions. For instance, clinical trials in patients with asthma have demonstrated that saffron supplementation (eg, 100 mg/day) can significantly improve clinical symptoms and lung function (38). Although this study used a dose of 30 mg twice daily, the consistent improvement across respiratory diseases suggests broader therapeutic potential for saffron in modulating pulmonary-related distress.

The clinical improvements observed in this study are supported by preclinical evidence. Animal studies have shown that saffron and its constituents, particularly crocin and safranal, exert bronchodilatory effects by modulating muscarinic and histamine H_1_ receptors. Furthermore, saffron has demonstrated antitussive properties through inhibition of inflammatory signaling pathways, such as nuclear factor κB, and reduction of Th2 cytokines, such as interleukin 4 and interleukin 5, which are key drivers of pulmonary inflammation. These mechanistic insights from preclinical models suggest that the observed reduction in respiratory distress in patients with sarcoidosis may be attributed to saffron’s ability to modulate both smooth muscle tone and systemic inflammatory cascades. In addition, modulation of neurotransmitters, particularly enhancement of γ-aminobutyric acid-mediated and serotonergic signaling, may further explain the observed improvements in sleep architecture and fatigue (39).

A primary finding of this study was the substantial improvement in general sleep disturbances, as measured by the GSDS. The saffron group showed a statistically significant reduction in GSDS scores compared with the control group (adjusted mean difference, -14.60; 95% CI, -17.23 to -11.97; P < 0.001). Notably, this outcome had the largest effect size among all measured variables (partial η^2^ = 0.64), indicating a robust clinical impact. These results are consistent with previous research suggesting that saffron’s bioactive compounds, such as crocin and safranal, may enhance sleep quality by modulating neurotransmitters such as γ-aminobutyric acid and serotonin (39). The significant improvement in GSDS scores indicates that saffron supplementation effectively addressed the multifaceted sleep challenges experienced by patients with pulmonary sarcoidosis.

In this study, the saffron group had a significant reduction in ESS scores compared with the control group (adjusted mean difference, -2.46; 95% CI, -3.32 to -1.61; P < 0.001), indicating a notable decrease in daytime drowsiness. Hinz et al. (37) reported that excessive daytime sleepiness affects approximately 50% of patients with sarcoidosis, with no specific treatments identified for this symptom. The present findings suggest that saffron may be a promising option for managing excessive daytime sleepiness in this population. Current pharmacological treatments for excessive daytime sleepiness, primarily for narcolepsy, include US Food and Drug Administration-approved medications such as modafinil, armodafinil, dextroamphetamine, mixed amphetamine/dextroamphetamine, methylphenidate, sodium oxybate, solriamfetol, and pitolisant, as well as off-label options that target either the underlying cause or the symptoms of excessive sleepiness (40). Given the prevalence of sleep disturbances in pulmonary sarcoidosis, the present results indicate that saffron supplementation may improve both sleep quantity and quality in these patients, offering a potential new approach for managing these symptoms (32).

The SF-12 questionnaire was used to assess health-related quality of life. The MCS reflects psychological well-being and social functioning, whereas the PCS evaluates general health and physical abilities (30, 41). The present findings showed that saffron supplementation significantly improved both components, with a more substantial improvement in the mental component. The between-group difference was 3.99 points for the MCS (P = 0.003) and 2.17 points for the PCS (P = 0.03). The PROMIS global physical health score also improved significantly (adjusted mean difference, 1.65; P < 0.001), further supporting the positive impact of saffron on patients’ perceived physical health. These results are particularly important given that a systematic review by Vis et al. (42) highlighted the current lack of effective pharmacological treatments for impaired quality of life and fatigue in patients with sarcoidosis.

These findings are consistent with previous research showing the efficacy of saffron in improving sleep quality and mood among individuals with insomnia, anxiety, and depression. However, research specifically targeting patients with pulmonary sarcoidosis remains scarce. By providing evidence of saffron’s efficacy in this population, in which chronic inflammation, sleep disturbances, and fatigue are closely intertwined, this study fills an important gap in the literature and supports the use of *Crocus sativus* as a viable adjunctive intervention to enhance quality of life.

A major strength of this study is its randomized controlled, single-blind design, which minimized potential measurement bias. In addition, the use of multiple validated tools (PSQI, GSDS, ESS, FAS, PROMIS, and SF-12) enabled a comprehensive evaluation of saffron’s effects on various dimensions of sleep and fatigue. The adequate sample size also supports the applicability of these findings to a broader population of patients with pulmonary sarcoidosis.

### 5.1. Clinical Implications

Saffron supplementation may offer a safe and accessible complementary option for improving sleep quality and reducing fatigue in patients with pulmonary sarcoidosis. The large effect size observed for general sleep disturbances (partial η^2^ = 0.64) underscores the potent impact of saffron on sleep outcomes in this population. These findings suggest that its clinical benefits are robust and reliable for the management of sarcoidosis-associated symptoms.

### 5.2. Limitations and Implications for Future Research

Several limitations should be acknowledged. First, the absence of a placebo group and the single-blind design may have introduced bias in self-reported outcomes, although the use of multiple validated tools helped mitigate this risk. Second, the 60-day intervention period limits insights into the long-term sustainability of saffron’s benefits. Third, the study population was relatively homogeneous and focused primarily on pulmonary sarcoidosis, which may limit the generalizability of the findings to other systemic manifestations of the disease. Future research should use double-blind, placebo-controlled designs with larger, more diverse cohorts and longer follow-up periods. In addition, studies investigating the biological mechanisms underlying saffron’s effects, particularly its impact on neuroinflammation and oxidative stress biomarkers, will be important for fully elucidating its therapeutic potential.

### 5.3. Conclusions

This study demonstrates that saffron supplementation (30 mg twice daily) significantly improves sleep quality, reduces daytime sleepiness, and alleviates fatigue in patients with pulmonary sarcoidosis. Given its favorable safety profile and the current lack of effective pharmacological treatments for these symptoms, saffron represents a promising adjunctive therapy to enhance quality of life in this population. Although further large-scale, placebo-controlled studies are needed to establish long-term efficacy and optimal dosing, these findings support the integration of saffron as a supportive intervention in the clinical management of sarcoidosis.

## Data Availability

The data presented in this study are uploaded during submission as a supplementary file and are openly available for readers upon request.

## Clinical Trial Registration Code:

IRCT20250224064834N1.
